# Lifetime Pesticide Use and Telomere Shortening among Male Pesticide Applicators in the Agricultural Health Study

**DOI:** 10.1289/ehp.1206432

**Published:** 2013-06-07

**Authors:** Lifang Hou, Gabriella Andreotti, Andrea A. Baccarelli, Sharon Savage, Jane A. Hoppin, Dale P. Sandler, Joseph Barker, Zhong-Zheng Zhu, Mirjam Hoxha, Laura Dioni, Xiao Zhang, Stella Koutros, Laura E. Beane Freeman, Michael C. Alavanja

**Affiliations:** 1 Department of Preventive Medicine, and; 2 The Robert H. Lurie Comprehensive Cancer Center, Feinberg School of Medicine, Northwestern University, Chicago, Illinois, USA; 3 Division of Cancer Epidemiology and Genetics, National Cancer Institute, National Institutes of Health (NIH), Department of Health and Human Services (DHHS), Bethesda, Maryland, USA; 4 Exposure, Epidemiology and Risk Program, Harvard School of Public Health, Boston, Massachusetts, USA; 5 Epidemiology Branch, National Institute of Environmental Health Sciences, NIH, DHHS, Research Triangle Park, North Carolina, USA; 6 IMS, Silver Spring, Maryland, USA; 7 Department of Oncology, No. 113 Hospital of People’s Liberation Army, Ningbo, China; 8 Center of Molecular and Genetic Epidemiology, Department of Environmental and Occupational Health, University of Milan, Milan, Italy; 9 Istituto di Ricovero e Cura a Carattere Scientifico Fondazione Ospedale Maggiore Policlinico, Mangiagalli e Regina Elena di Milano, Milan, Italy

**Keywords:** Agricultural Health Study, cancer-free subjects, occupational exposures, pesticides, telomere length

## Abstract

Background: Telomere length (TL) in surrogate tissues may be influenced by environmental exposures.

Objective: We aimed to determine whether lifetime pesticides use is associated with buccal cell TL.

Methods: We examined buccal cell TL in relation to lifetime use of 48 pesticides for 1,234 cancer-free white male pesticide applicators in the Agricultural Health Study (AHS), a prospective cohort study of 57,310 licensed pesticide applicators. Participants provided detailed information on lifetime use of 50 pesticides at enrollment (1993–1997). Buccal cells were collected from 1999 to 2006. Relative telomere length (RTL) was measured using quantitative real-time polymerase chain reaction. We used linear regression modeling to evaluate the associations between specific pesticides and the logarithm of RTL, adjusting for age at buccal cell collection, state of residence, applicator license type, chewing tobacco use, and total lifetime days of all pesticide use.

Results: The mean RTL for participants decreased significantly in association with increased lifetime days of pesticide use for alachlor (*p* = 0.002), 2,4-dichlorophenoxyacetic acid (2,4-D; *p* = 0.004), metolachlor (p = 0.01), trifluralin (*p* = 0.05), permethrin (for animal application) (*p* = 0.02), and toxaphene (*p* = 0.04). A similar pattern of RTL shortening was observed with the metric lifetime intensity-weighted days of pesticide use. For dichlorodiphenyltrichloroethane (DDT), we observed significant RTL shortening for lifetime intensity-weighted days (*p* = 0.04), but not for lifetime days of DDT use (*p* = 0.08). No significant RTL lengthening was observed for any pesticide.

Conclusion: Seven pesticides previously associated with cancer risk in the epidemiologic literature were inversely associated with RTL in buccal cell DNA among cancer-free pesticide applicators. Replication of these findings is needed because we cannot rule out chance or fully rule out bias.

## Introduction

Pesticides are widely used in the United States and worldwide and are pervasive in our environment. Several pesticides have been associated with various cancers in experimental studies [summarized by the U.S. Environmental Protection Agency (U.S. EPA 2012)] and in epidemiologic studies of farmers (Blair and Freeman 2009) and pesticide manufacturing workers (Acquavella et al. 1996; Fryzek et al. 1997; Kogevinas et al. 1997; Leet et al. 1996) and among register pesticide applicators, including the Agricultural Health Study (AHS) cohort, one of the largest prospective studies of pesticide applicators (Alavanja et al. 1996).

The mechanisms by which pesticides may be linked to cancers in humans are unclear. Potential mechanisms include oxidative stress, DNA damage, chromosome aberration, immune response abnormality, and chronic inflammation (Casale et al. 1998; Hooghe et al. 2000; Stiller-Winkler et al. 1999; Undeger and Basaran 2005). These biological processes are also involved in telomere shortening (von Zglinicki 2002). The genetic integrity of the genome is maintained, in part, by the architecture of telomeres (Artandi et al. 2000; Meeker et al. 2004). Telomeres typically shorten with each cell division. When telomeres reach a critical length, cellular apoptosis or senescence is triggered. Cancer cells bypass these pathways and continue to divide despite the presence of chromosomal instability (Blasco 2005). Human epidemiologic investigations, mostly from case–control studies, have suggested that telomere length (TL) in surrogate tissues (i.e., blood or buccal cells) is associated with some but not all cancers (Broberg et al. 2005; Hou et al. 2009; Jang et al. 2008; McGrath et al. 2007; Risques et al. 2007; Shao et al. 2007; Widmann et al. 2007). While most studies have reported that shorter telomeres in surrogate tissue are positively associated with cancer [as reviewed by Ma et al. (2011)and Wentzensen et al. (2011)], longer telomeres have been associated with cancer in some studies and reviews (Gramatges et al. 2010; Han et al. 2009; Lan et al. 2009; Shen et al. 2011; Svenson et al. 2009).

The potential effects of pesticide exposure on TL in surrogate tissues have not been well characterized, although associations with telomere shortening have been reported for occupational exposures (Eshkoor et al. 2011) and persistent organic pollutants (Shin et al. 2010). In the present study, we examined whether lifetime use of 48 pesticides is associated with telomere shortening in buccal cell DNA from 1,234 white male cancer-free licensed pesticide applicators participating in the AHS.

## Materials and Methods

*Study population*. A detailed description of the AHS has been published (Alavanja et al. 1996). Briefly, 57,310, or 82%, of pesticide applicators seeking pesticide licensing in the U.S. states of Iowa and North Carolina were enrolled between 13 December 1993 and 31 December 1997. All data used in these analyses were based on AHS data release P1REL0506.01. Enrolled participants were licensed private pesticide applicators (mostly farmers) residing in Iowa and North Carolina and commercial applicators residing in Iowa. All pesticide applicators completed an enrollment questionnaire that inquired about ever/never use of 50 pesticides (National Institutes of Health 2013) as well as the duration (years) and frequency (average days per year) of use for 22 of these pesticides. In addition, 44% of the participants completed a second take-home questionnaire at enrollment that inquired about the duration and frequency of use of the remaining 28 pesticides. Approximately 5 years after enrollment, participants completed a follow-up phone interview to collect additional pesticide use and medical history information and were asked to provide a mouthwash rinse sample for extraction of DNA from buccal cells. Participants who agreed to provide buccal cells were sent a buccal cell collection kit and a postage-paid, padded envelope. Informed consent for buccal cell collection and buccal cell analyses associated with potential carcinogenic risk assessment was obtained at the time of collection, and the study protocol and informed consent was reviewed by all relevant institutional review boards. A total of 36,342 (63%) participants completed the follow-up interview, and 20,421 (56%) returned buccal cells. No meaningful difference in demographics was observed between those who donated buccal cells and those who did not (Engel et al. 2002).

As part of a nested case–control study of prostate cancer, 1,372 participants who had no history of prostate cancer (controls) and were > 40 years of age at the time of buccal cell collection were initially selected for TL measurement. Of these, 1,234 met inclusion criteria for the TL analysis. Participants excluded from the analysis were 115 men who had been diagnosed with any cancer prior to or within 3 years of buccal cell collection, 14 men who donated a buccal cell sample but did not provide a completed written consent at the time of the present analysis, and 9 men who were nonwhite. The present analysis was limited to white applicators because of the small numbers of nonwhite applicators in the AHS cohort (Alavanja et al. 1996).

*Pesticide exposure*. Two pesticide exposure metrics were used. Pesticide use was evaluated as both lifetime days of pesticide use (years of use × days per year) and lifetime intensity-weighted days of pesticide use (lifetime exposure days × intensity score). The intensity score was computed from an algorithm that took into account exposure-modifying factors such as application method and protective equipment use (Coble et al. 2011).

*Buccal cell collection*. Buccal cells were collected from 1999 through 2006 using a mouthwash “swish and spit” collection technique (García-Closas et al. 2001). Buccal cells collection vials were returned to the National Cancer Institute (NCI) repository via a postage-paid, padded envelope. All samples were stored at the NCI repository at –80°C. DNA from buccal cells was extracted using the Wizard® Genomic DNA Purification Kit (Promega Corp., Madison, WI, USA).

*TL measurements*. Relative telomere length (RTL) in buccal DNA was measured at the Laboratory of Environmental Epigenetics, Center of Molecular and Genetic Epidemiology, Milan University, Italy, using quantitative real-time polymerase chain reaction (RT-qPCR) as described previously (Cawthon 2002). Briefly, this method measures RTL in genomic DNA by determining the ratio of telomere repeat copy number (T) to single copy gene (S) (*36B4* gene located on chromosome 12, which encodes acidic ribosomal phosphoprotein P0) copy number (T/S ratio) in individual samples relative to a reference pooled DNA (Boulay et al. 1999). The reference pooled DNA was created using samples from 60 participants randomly selected from the population sample selected for this study and was used to generate a fresh standard curve, ranging from 0.25 to 8 ng/µL in every T and S RT-qPCR run [see Supplemental Material, Figure S1 (http://dx.doi.org/10.1289/ehp.1206432)]. All samples were successfully run in duplicate with a 100% completion rate. The inter-batch variability [coefficient of variation (CV)] in this study was 8.1%. The primer sequences and concentrations were GGTT​TTTG​AGGG​TGAG​GGTG​AGGG​TGAG​GGT (270 nM) and TCCC​GACT​ATCC​CTAT​CCCT​ATCC​CTAT​CCCT​ATCC​-CTA (900 nM) for telomere; and CAGC​AAGT​GGGA​AGGT​GTAA​TCC (300 nM) and CCCA​TTCT​ATCA​TCAA​CGGG​TACA​A (500 nM) for human beta-globin.

The T (telomere) RT-qPCR mix was iQ SYBR Green Supermix (Bio-Rad, Hercules, California, USA) 1×, tel1b 100 nM, tel2b 900 nM, DMSO 1%, EDTA 1×. The S (human beta-globin) RT-qPCR mix was iQ SYBR Green Supermix (Bio-Rad) 1×, hbg1 300 nM, hbg2 700 nM, DMSO 1%, DTT 2,5 mM, EDTA 1×. We used the RT-qPCR primer sets previously described by McGrath et al. (2007). We used pooled DNA from 20 referents (500 ng for each sample), randomly selected from samples of this same study, to create a fresh standard curve, ranging from 8 ng/μL to 0.5 ng/μL, at every T and S RT-qPCR run. All samples contained *Eschericheria coli* DNA heated at 96°C × 10 min and cooled at room temperature. 15 ng of DNA samples was added to each reaction (final volume, 20 μL). All RT-qPCRs were performed on a DNA Engine thermal cycler Chromo4 (Bio-Rad). The thermal cycling profile for both amplicons started with a 95°C incubation for 3 min to activate the hot-start iTaq DNA polymerase. The T RT-qPCR continued with 25 cycles at 95°C for 15 sec, and anneal/extend at 54°C for 49 sec. The S RT-qPCR continued with 35 cycles at 95°C for 15 sec, anneal at 58°C for 1s, extend at 72°C for 15 sec. At the end of each reaction, a melting curve was used for both T and S RT-qPCRs. All samples were run in triplicates.

*Statistical analysis*. The means of all three RTL measurements were used in the statistical analyses. Mean RTL values reported in Table 1 are the arithmetic mean stratum-specific estimates of RTL ± SD. Because RTL and both pesticide exposure metrics (i.e., lifetime-days and lifetime intensity-weighted days) had right-skewed frequency distributions, a natural logarithm transformation was applied to RTL and to both exposure metrics. Linear regression models were used to estimate the change in RTL with increasing pesticide use on a continuous scale (i.e., lifetime-days and lifetime intensity-weighted days). For presentation purposes, we also calculated the mean RTL for the reference group (i.e., no use of the pesticide) and each tertile of lifetime intensity-weighted days of pesticide use; however, the continuous measure was used in the analysis for testing statistical significance. Two pesticides (trichlorofon and ziram) had small numbers of exposed participants (*n* < 20) and were dropped from the analyses. *p*-Values for the linear regression coefficient between each pesticide and RTL, showing the continuous change in RTL with increasing days of use, were adjusted for age at buccal cell sample collection (as a continuous variable), state of residence (Iowa vs. North Carolina), applicator license type (private, commercial), use of chewing tobacco regularly for 6 months or longer (yes vs. no) and total lifetime-days of all pesticide use (continuous). Further adjusting the linear regression model for body mass index (BMI) (continuous), alcohol consumption (none vs. any; or none, < 3, or ≥ 3 drinks per week), smoking (pack-years, current vs. never, ever vs. never), and self-reported cardiovascular disease, diabetes, and high blood pressure produced comparable results (data not shown), and these factors were not included in our final models. All tests were two-sided and *p* ≤ 0.05 was considered significant. All statistical analyses were conducted using AHS Data Release, version P1REL0506.01, and SAS, version 9.2 (SAS Institute Inc., Cary, NC, USA).

**Table 1:** Mean RTL by selected characteristics of the cancer-free study population (*n* = 1,234).*^a^*

Characteristic	Group	*n*	RTL (mean ± SD)^*b*^	*p*-Value
Age at buccal cell collection (years)^*c*^	41–62	312	1.25 ± 0.34
	63–67	295	1.21 ± 0.39
	68–72	333	1.18 ± 0.33
	73–92	294	1.18 ± 0.38	0.003
BMI	18.65–25.66	392	1.20 ± 0.34
	25.67–28.60	394	1.21 ± 0.38
	28.61–46.98	385	1.21 ± 0.36	0.93
Applicator type	Private	1,184	1.21 ± 0.36
	Commercial	50	1.08 ± 0.43	0.01
State of residence	Iowa	831	1.19 ± 0.32
	North Carolina	403	1.24 ± 0.43	0.03
Education	≤ High school	805	1.20 ± 0.37
	> High school	406	1.21 ± 0.36	0.71
Smoking status	Never	563	1.19 ± 0.32
	Former	562	1.20 ± 0.38
	Current	97	1.25 ± 0.42	0.63
Pack-years of smoking	No	563	1.19 ± 0.32
	< 20	372	1.20 ± 0.32
	≥ 20	258	1.21 ± 0.47	0.90
Chewing tobacco use	No	1,087	1.19 ± 0.36
	Yes	147	1.27 ± 0.34	0.01
Alcohol drinking	No	516	1.22 ± 0.39
	Yes	663	1.19 ± 0.31	0.10
	Low (< 3 drinks/week)	346	1.17 ± 0.34
	High (≥ 3 drinks/week)	317	1.20 ± 0.34	0.25
Cardiovascular disease	No	907	1.21 ± 0.36	0.88
	Yes	326	1.21 ± 0.38
Diagnosis of diabete	No	1,167	1.20 ± 0.36
	Yes	66	1.20 ± 0.33	0.78
High blood pressure	No	607	1.17 ± 0.32
	Yes	225	1.19 ± 0.33	0.22
Family history of any cancers	No	548	1.20 ± 0.33
	Yes	613	1.24 ± 0.39	0.88
Use of any pesti­cides	Low (≤ 236 days)	582	1.21 ± 0.35
	High (> 236 days)	606	1.20 ± 0.37	0.48
^***a***^*p*-Values from linear regression on ln-transformed RTL (continuous) adjusted for age at buccal cell sample collection (continuous) for all characteristics other than age. ^***b***^Arithmetic mean for the stratum-specific estimate of RTL ± SD. ^***c***^Median age at buccal cell collection, 68 years.				

## Results

Regression analysis *p*-values for model coefficients of ln-transformed pesticide exposures adjusted for age at buccal cell collection are shown according to selected characteristics in Table 1. RTL was negatively associated with age at buccal cell sample collection (*p* = 0.003). Commercial pesticide applicators had shorter RTL (mean = 1.08) than private applicators (mean = 1.21, *p* = 0.01). The mean RTL in Iowa applicators (1.19) was significantly shorter than the mean value in North Carolina applicators (mean = 1.24, *p* = 0.03). The mean RTL was significantly longer for men who used chewing tobacco for ≥ 6 months (mean = 1.27) than men who did not (mean = 1.19, *p* = 0.01). Self-reported education, BMI, smoking status, pack-years smoked, alcohol consumption amount, family history of any cancers, and cardiovascular disease, diabetes, and high blood pressure were not significantly associated with RTL. It is worth noting that although RTL among current smokers is not statistically significant (overall *p* > 0.05), both current smokers and those chewing tobacco had longer telomeres. All study participants applied some pesticides, but a comparison of those with more than the median number of applications days of total pesticide use versus those with less than the median number did not show any difference in RTL.

Among the 48 pesticides examined, increasing lifetime-days of pesticide use for 6 pesticides [alachlor, metolachlor, trifluralin, 2,4-dichlorophenoxyacetic acid (2,4-D), permethrin, and toxaphene] were significantly (*p* < 0.05) associated with decreases in RTL after adjusting for age at buccal cell collection, state of residence, license type, use of chewing tobacco, and total pesticide-application days [Table 2; see also Supplemental Material, Table S1 (http://dx.doi.org/10.1289/ehp.1206432) for results for the other pesticides evaluated]. Of these, four were herbicides [alachlor (*p* = 0.002), metolachlor (*p* = 0.01), trifluralin (*p* = 0.05), and 2,4-D (*p* = 0.004)], and two were insecticides [permethrin (*p* = 0.02) and toxaphene (*p* = 0.04)]. No significant RTL lengthening was observed for any pesticide (Table 2; see also Supplemental Material, Table S1).

**Table 2:** Lifetime pesticide-use days, intensity-weighted lifetime pesticide-use days, and RTL.

Pesticide (class)	Pesticide use (continous)	Lifetime days			Lifetime intensity-weighted days		
		*n*	RTL mean ± SD	*p*_trend_^*a*^(β ± SE)	*n*	RTL mean ± SD	*p*_trend_^*a*^(β ± SE)
Herbicides
Alachlor (chloroacetanilde)	No use	466	1.24 ± 0.41		466	1.24 ± 0.41
	Low	225	1.18 ± 0.31		221	1.14 ± 0.28
	Medium	215	1.18 ± 0.32		219	1.19 ± 0.36
	High	219	1.16 ± 0.33	0.002 (–0.011 ± 0.010)	219	1.18 ± 0.32	0.005 (–0.007 ± 0.002)
Metolachlor (acetamide)	No use	622	1.23 ± 0.38		622	1.23 ± 0.38
	Low	223	1.17 ± 0.31		165	1.18 ± 0.32
	Medium	112	1.18 ± 0.31		164	1.18 ± 0.33
	High	157	1.16 ± 0.37	0.01 (–0.013 ± 0.004)	163	1.16 ± 0.34	0.01 (–0.006 ± 0.002)
Trifluralin (dinitroaniline)	No use	515	1.23 ± 0.36		515	1.23 ± 0.36
	Low	249	1.20 ± 0.39		200	1.17 ± 0.42
	Medium	191	1.17 ± 0.33		199	1.19 ± 0.33
	High	159	1.16 ± 0.33	0.05 (–0.008 ± 0.004)	199	1.18 ± 0.31	0.06 (–0.004 ± 0.002)
2,4-D (phenoxyacid)	No use	194	1.27 ± 0.48		194	1.27 ± 0.48
	Low	366	1.21 ± 0.36		334	1.21 ± 0.37
	Medium	320	1.21 ± 0.31		338	1.18 ± 0.32
	High	318	1.16 ± 0.32	0.004 (–0.012 ± 0.004)	330	1.18 ± 0.31	0.02 (–0.006 ± 0.002)
Insecticide
DDT (organochlorine)	No use	428	1.21 ± 0.34		428	1.21 ± 0.34
	Low	153	1.13 ± 0.33		126	1.10 ± 0.33
	Medium	97	1.13 ± 0.32		124	1.15 ± 0.30
	High	121	1.16 ± 0.30	0.08 (–002 ± 0.006)	121	1.18 ± 0.31	0.03 (–0.006 ± 0.003)
Permethrin (poultry/livestock) (pyrethroid)	No use	1,021	1.21 ± 0.37		1,021	1.21 ± 0.37
	Low	36	1.17 ± 0.27		34	1.16 ± 0.26
	Medium	40	1.16 ± 0.28		35	1.12 ± 0.29
	High	28	1.10 ± 0.26	0.02 (–0.018 ± 0.008)	33	1.14 ± 0.26	0.02 (–0.010 ± 0.004)
Toxaphene (organochlorine mixture)	No use	679	1.18 ± 0.33		679	1.18 ± 0.33
	Low	60	1.13 ± 0.39		43	1.08 ± 0.39
	Medium	31	1.15 ± 0.28		43	1.22 ± 0.34
	High	37	1.14 ± 0.27	0.04 (–0.017 ± 0.009)	42	1.11 ± 0.25	0.05 (–0.008 ± 0.004)
^***a***^*p*-Value of regression coefficient for ln-RTL (continuous) regressed on ln-transformed lifetime days of pesti­cide use (continuous) or ln-transformed lifetime intensity weighted days of pesti­cide use (continuous), adjusted for age at buccal collection (continuous), state (Iowa vs. North Carolina), license types (private vs. commercial), regular use of chewing tobaccofor ≥ 6 months (yes vs. no), total pesti­cide exposure days (continuous). RTL mean is the arithmetic mean for the stratum-specific RTL by pesti­cide-use group.							

Comparable results were observed with lifetime intensity-weighted days of pesticide use (Table 2) for alachlor (*p* = 0.005), metolachlor (*p* = 0.01), permethrin (*p* = 0.02), 2,4-D (*p* = 0.02), and toxaphene (*p* = 0.05). RTL also decreased with increasing use of trifluralin (*p* = 0.06), but the association was not statistically significant. A statistically significant negative association with RTL was estimated for intensity-weighted lifetime-days of dichlorodiphenyltrichloroethane (DDT) use (*p* = 0.03), but not for lifetime-days of DDT use (*p* = 0.08). Adjustment for total days of pesticide use and for use of chewing tobacco did not affect the associations with pesticides in any meaningful way.

## Discussion

Seven pesticides were negatively associated with RTL in buccal cell DNA among cancer-free pesticide applicators > 40 years of age. Increasing lifetime-days of use of six pesticides used in agriculture (alachlor, metolachlor, trifluralin, 2,4-D, permethrin, and toxaphene) were associated with significantly shorter telomeres after controlling for age at buccal cell collection, state of residence, license type, use of chewing tobacco, and total pesticide use days. Associations were similar between lifetime-days and intensity-weighted days of use of alachlor, metolachlor, 2,4-D, permethrin, and toxaphene. RTL also decreased with increasing lifetime intensity-weighted days of use of trifluralin, but the association was not statistically significant. For DDT, RTL shortening was significant in association with lifetime intensity-weighted days, but not lifetime-days of use.

The only study reporting data on pesticides and TL found that high levels of exposure to persistent organic pollutants (POPs), including organochlorine (OC) pesticides, polychlorinated biphenyls (PCBs), and polybrominated diphenylethers (PBDEs) were associated with decreased RTL in peripheral blood leukocyte DNA, yet low levels of exposure were associated with increased RTL in an apparently healthy Korean population (Shin et al. 2010). However, because of the limited sample size, and the very small numbers of observations at higher levels of exposure, more studies are needed to confirm the observation.

We observed that use of seven pesticides, as estimated by one or more exposure metrics, was associated with significantly decreasing RTL. Pesticide use has been noted to cause oxidative stress in humans (Honda et al. 2001; Houben et al. 2008; Meeker et al. 2004; von Zglinicki 2002), and telomere shortening has been associated with cumulative oxidative stress (Houben et al. 2008; von Zglinicki 2002). Telomeres are remarkably sensitive to damage by oxidative stress because of the high guanine content in specific telomere sequences and the deficiency in the repair of single-strand breaks (Honda et al. 2001; Meeker et al. 2004; von Zglinicki 2002). Pesticide exposure may also lead to telomere shortening by causing inflammation. Figgs et al. (2000) reported that urinary 2,4-D concentration was associated with an increased lymphocyte replicative index, a cell proliferation biomarker. The replicative index for lymphocytes was higher among applicators than non-applicators and higher among applicators after spraying than before spraying. Telomeric DNA is dynamic, and TL typically shortens with each cell division (Hou et al. 2012). Therefore, the increased lymphocyte replicative index associated with the use of 2,4-D could be associated with inflammation because extensive cell proliferation and clonal expansion is an essential part of an inflammatory response (Hodes et al. 2002).

Of the seven pesticides associated with telomere shortening, four were herbicides and three were insecticides. The use of these chemicals was not strongly correlated with each other in our sample (the range of correlation coefficients between the seven pesticides varied from 0.1 to 0.24). These pesticides belong to different chemical classes and there were no chemical or functional classes where all pesticides were linked to RTL shortening.

Alachlor has been shown to induce chromosomal aberrations in mouse bone marrow cells (Meisner et al. 1992) and to cause chromosomal damage in *in vitro* experimental studies using Chinese hamster ovary cells (Lin et al. 1987), which may be related to TL shortening. In the AHS, a positive association between alachlor and the incidence of lymphohematopoietic cancers was found among applicators (Lee et al. 2004), but no excess cancer risk was observed in a study of alachlor manufacturing workers (Acquavella et al. 2004).

Metolachlor has been associated with lung cancer in the AHS (Alavanja et al. 2004), and the U.S. EPA (1995) classifies metolachlor as a possible human carcinogen (U.S. EPA group C). Trifluralin has been associated with colon cancer in the AHS (Kang et al. 2008), and the U.S. EPA (1996) classifies trifluralin as a possible human carcinogen (U.S. EPA group C). 2,4-D has been associated with prostate cancer in a large case–control study in British Columbia (Band et al. 2011), but not in the AHS cohort (Koutros et al. 2013), and with non-Hodgkin lymphoma (NHL) in a number of studies (Burns et al. 2011; Hoar et al. 1986; McDuffie et al. 2001; Miligi et al. 2006). The U.S. EPA (2005) classifies 2,4-D as not classifiable (U.S. EPA group D) with regard to human carcinogenicity, and the International Agency for Research on Cancer (IARC 2001) classifies the chlorphenoxy herbicide group as possibly carcinogenic to humans (IARC group 2B). Permethrin has been associated with multiple myeloma in the AHS (Rusiecki et al. 2009). In laboratory studies, treatment with a high dose of permethrin induced significant lymphocyte DNA damage in a rat model (Gabbianelli et al. 2004). The IARC (2001) considers DDT and toxaphene possible human carcinogens (IARC group 2B). In a recent Danish study conducted within a prospective cohort, prediagnostic adipose concentrations of DDT demonstrated a significant positive monotonic dose–response trend with NHL incidence (Brauner et al. 2012).

The present study is unique in that we have a relatively large population of licensed pesticide applicators who provided reliable information regarding their pesticide application history (Blair et al. 2002; Coble et al. 2011). We examined the relationship between cumulative lifetime use of specific pesticides and RTL in a cancer-free population (i.e., the study participants had no cancer diagnoses). Furthermore, we were able to adjust for potential confounding factors related to RTL. In the AHS, *a priori*–derived algorithm scores that incorporated several exposure determinants were used to predict pesticide exposure intensity. These algorithm scores have been shown to predict urinary pesticides levels (Coble et al. 2011; Thomas et al. 2010). The significant decrease in RTL with age that we observed was expected (Blasco 2005) based on the literature, adding confidence to our pesticide-related findings. Most pesticides used by applicators are mixtures of one active ingredient and several “inerts” that are not pesticides, and there are some products that contain more than one active ingredient. The co-occurrence of active ingredients is an intractable problem for an epidemiologic study assessing a lifelong occupational history of pesticide use. This problem, however, is less common than the concern we were able to address, which is that over a lifetime applicators may use more than one product for the same or different crops. In this study we controlled for the total pesticide-exposure days and found no meaningful change in the associations, although confounding by specific pesticides cannot be ruled out. Although potential confounding from other occupational exposures is possible, the magnitude of bias due to this confounding was reported to be minimal in the AHS and not likely to be associated with pesticide exposures (Coble et al. 2002). The fact that other lifestyle factors (e.g., smoking and alcohol drinking) and chronic health conditions (e.g., cardiovascular disease, high blood pressure) were not significantly associated with TL in our population may be a result of our population being more physically active and older than in some previous studies. Physical activity has been shown to increase TL (Du et al. 2012) and TL seems to be less influenced by lifestyle factors in older populations (Tiainen et al. 2012). Future studies with increased statistical power will be needed to assess this issue more completely.

In this study, we used buccal cell DNA for TL measurement collected at a single point in time after exposure assessment was made. Buccal cells can be easily and inexpensively collected and are therefore a convenient resource for evaluating TL-related disease risks. Future studies may benefit from collecting buccal cells at multiple points in time to assess the rate at which telomeres are shortened. Our results should, however, be interpreted with caution because TL in buccal DNA may be different from that in diseased tissue or blood DNA (Prescott et al. 2012; Wong et al. 2011). We observed that cumulative lifetime use of some pesticides is associated with telomere shortening in buccal cell DNA, although we cannot rule out the possibility of bias due to uncontrolled confounding, or associations due to chance because of the multiple comparisons that were made. Our findings suggest that specific pesticides may contribute to telomere shortening and may serve as a mechanism for development of some diseases. Given that this is one of the first studies of pesticide exposure and TL, we view this work as hypothesis generating rather than hypothesis testing.

## Supplemental Material

(565 KB) PDF
