# Supplementary material for: Lifetime Pesticide Use and Telomere Shortening among Male Pesticide Applicators in the Agricultural Health Study
Source: Environ Health Perspect. 2013 Jun 7;121(8):919–24. doi: 10.1289/ehp.1206432 (PMC3734498; doi:10.1289/ehp.1206432)

# Supplemental Material

# Lifetime Pesticide Use and Telomere Shortening among Male Pesticide Applicators in the Agricultural Health Study

Lifang Hou, Gabriella Andreotti, Andrea A. Baccarelli, Sharon Savage, Jane A. Hoppin, Dale P. Sandler, Joseph Barker, Zhong-Zheng Zhu, Mirjam Hoxha, Laura Dioni, Xiao Zhang, Stella Koutros, Laura E. Beane Freeman, and Michael C. Alavanja

**Table S1.** Lifetime pesticides use days, intensity-weighted lifetime pesticide use days and relative telomere length

| | | Lifetime Days | | | Lifetime Intensity-weighted Days | | |
|---|---|---|---|---|---|---|---|
| **Pesticide (class)** | **Pesticide Use (continuous)** | **No.** | **RTL Mean ± SD** | **P for trend[a] (β± SE)** | **No.** | **RTL Mean ± SD** | **P for trend[b] (β±SE)** |
| **Herbicides** | | | | | | | |
| **Atrazine (triazine)** | Not exp | 320 | 1.21 ± 0.35 | | 320 | 1.21 ± 0.35 | |
| | Low | 342 | 1.21± 0.39 | | 297 | 1.20 ± 0.41 | |
| | Medium | 249 | 1.19 ± 0.34 | | 292 | 1.21 ± 0.32 | |
| | High | 293 | 1.20 ± 0.34 | 0.54 (-0.002±0.004) | 294 | 1.19 ± 0.34 | 0.78 (-0.0006+0.002) |
| **Butylate (thiocarbamate)** | No use | 592 | 1.18 ± 0.34 | | 592 | 1.18 ± 0.34 | |
| | Low | 74 | 1.15 ± 0.31 | | 73 | 1.14 ± 0.29 | |
| | Medium | 75 | 1.18 ± 0.33 | | 73 | 1.18 ± 0.33 | |
| | High | 69 | 1.24 ± 0.35 | 0.41 (0.005±0.006) | 72 | 1.24 ± 0.36 | 0.42 (0.002±0.003) |
| **Chlorimuron-ethyl (pyrididine)** | No use | 586 | 1.19 ± 0.35 | | 586 | 1.19 ± 0.35 | |
| | Low | 140 | 1.16 ± 0.29 | | 81 | 1.17 ± 0.30 | |
| | Medium | 12 | 1.08 ± 0.25 | | 70 | 1.11 ± 0.30 | |
| | High | 73 | 1.18 ± 0.36 | 0.56 (-0.004±0.007) | 74 | 1.19 ± 0.33 | 0.48 (-0.002±0.003) |
| **Cyanazine (triazine)** | No use | 610 | 1.22 ± 0.39 | | 610 | 1.22 ± 0.39 | |
| | Low | 200 | 1.21 ± 0.35 | | 170 | 1.18 ± 0.35 | |
| | Medium | 160 | 1.17 ± 0.34 | | 169 | 1.20 ± 0.35 | |
| | High | 149 | 1.17 ± 0.30 | 0.33 (-0.004±0.004) | 169 | 1.17 ± 0.30 | 0.47 (-0.002±0.002) |
| **Dicamba (benzoic)** | No use | 510 | 1.23 ± 0.42 | | 510 | 1.23 ± 0.42 | |
| | Low | 270 | 1.17 ± 0.31 | | 201 | 1.16 ± 0.31 | |
| | Medium | 183 | 1.18 ± 0.34 | | 200 | 1.18 ± 0.34 | |
| | High | 149 | 1.17 ± 0.30 | 0.12 (-0.008±0.005) | 200 | 1.18 ± 0.29 | 0.16 (-0.004±0.003) |
| **EPTC (thiocarbamate)** | No use | 899 | 1.21 ± 0.37 | | 899 | 1.21 ± 0.37 | |
| | Low | 87 | 1.15 ± 0.34 | | 69 | 1.16 ± 0.36 | |
| | Medium | 52 | 1.18 ± 0.29 | | 70 | 1.17 ± 0.30 | |
| | High | 68 | 1.19 ± 0.30 | 0.56 (-0.004±0.006) | 68 | 1.18 ± 0.28 | 0.44 (-0.002±0.003) |
| **Glyphosate (phosphinic acid)** | No use | 291 | 1.19 ± 0.33 | | 291 | 1.19 ± 0.33 | |
| | Low | 391 | 1.19 ± 0.36 | | 307 | 1.18 ± 0.33 | |
| | Medium | 275 | 1.20 ± 0.32 | | 308 | 1.20 ± 0.36 | |
| | High | 254 | 1.21 ± 0.43 | 0.96 (-0.0002±0.004) | 304 | 1.23 ± 0.41 | 0.81 (0.0006±0.002) |

| Pesticide (class) | Pesticide Use (continuous) | Lifetime Days No. | Lifetime Days RTL Mean ± SD | Lifetime Days P for trend[a] (β± SE) | Lifetime Intensity-weighted Days No. | Lifetime Intensity-weighted Days RTL Mean ± SD | Lifetime Intensity-weighted Days P for trend[b] (β±SE) |
|---|---|---|---|---|---|---|---|
| **Imazethapyr (imidazolinone)** | No use | 669 | 1.20 ± 0.38 | | 669 | 1.20 ± 0.38 | |
| | Low | 198 | 1.17 ± 0.29 | | 148 | 1.16 ± 0.30 | |
| | Medium | 140 | 1.25 ± 0.30 | | 145 | 1.23 ± 0.35 | |
| | High | 97 | 1.19 ± 0.36 | 0.32 (0.006±0.006) | 141 | 1.20 ± 0.36 | 0.31 (0.003±0.003) |
| **Metribuzin (triazine)** | No use | 490 | 1.19 ± 0.35 | | 490 | 1.19 ± 0.35 | |
| | Low | 160 | 1.17 ± 0.32 | | 107 | 1.18 ± 0.34 | |
| | Medium | 72 | 1.19 ± 0.28 | | 106 | 1.14 ± 0.27 | |
| | High | 87 | 1.11 ± 0.33 | 0.31 (-0.007±0.006) | 106 | 1.14 ± 0.32 | 0.52 (-0.002±0.003) |
| **Paraquat (pyridine herbicide)** | No use | 666 | 1.17 ± 0.33 | | 666 | 1.17 ± 0.33 | |
| | Low | 83 | 1.19 ± 0.34 | | 50 | 1.11 ± 0.33 | |
| | Medium | 28 | 1.23 ± 0.40 | | 48 | 1.27 ± 0.33 | |
| | High | 33 | 1.26 ± 0.35 | 0.75 (0.003±0.009) | 46 | 1.27 ± 0.37 | 0.73 (0.001±0.004) |
| **Pendimethalin (dinitroaniline)** | No use | 548 | 1.18 ± 0.33 | | 548 | 1.18 ± 0.33 | |
| | Low | 142 | 1.18 ± 0.35 | | 91 | 1.16 ± 0.36 | |
| | Medium | 56 | 1.21 ± 0.32 | | 90 | 1.21 ± 0.34 | |
| | High | 72 | 1.16 ± 0.34 | 0.83 (-0.001±0.006) | 89 | 1.17 ± 0.33 | 0.99 (-0.00002±0.003) |
| **Petroleum oils (aliphatic hydrocarbon)** | No use | 612 | 1.18 ± 0.34 | | 612 | 1.18 ± 0.34 | |
| | Low | 67 | 1.17 ± 0.29 | | 62 | 1.14 ± 0.29 | |
| | Medium | 60 | 1.15 ± 0.28 | | 63 | 1.20 ± 0.27 | |
| | High | 58 | 1.24 ± 0.33 | 0.51 (0.003±0.005) | 60 | 1.22 ± 0.34 | 0.44 (0.002±0.003) |
| **2,4,5 trichlorophenoxy acetic acid (2,4,5 T; chlorophenoxy acetic acid)** | No use | 538 | 1.19 ± 0.34 | | 538 | 1.19 ± 0.34 | |
| | Low | 132 | 1.15 ± 0.30 | | 89 | 1.11 ± 0.30 | |
| | Medium | 52 | 1.14 ± 0.34 | | 90 | 1.19 ± 0.30 | |
| | High | 83 | 1.14 ± 0.31 | 0.26 (-0.025±0.029) | 88 | 1.13 ± 0.33 | 0.26 (-0.014±0.016) |
| **2,4,5 TP (chlorphenoxy acetic acid)** | No use | 734 | 1.18 ± 0.30 | | 734 | 1.18 ± 0.34 | |
| | Low | 37 | 1.16 ± 0.29 | | 24 | 1.18 ± 0.29 | |
| | Medium | 15 | 1.23 ± 0.30 | | 25 | 1.18 ± 0.33 | |
| | High | 20 | 1.11 ± 0.23 | 0.81 (-0.003±0.011) | 23 | 1.12 ± 0.21 | 0.80 (-0.001±0.005) |
| **Insecticide** | | | | | | | |
| **Aldicarb (carbamate )** | No use | 770 | 1.18 ± 0.33 | | 770 | 1.18 ± 0.33 | |

| Pesticide (class) | Pesticide Use (continuous) | Lifetime Days No. | Lifetime Days RTL Mean ± SD | Lifetime Days P for trend[a] (β± SE) | Lifetime Intensity-weighted Days No. | Lifetime Intensity-weighted Days RTL Mean ± SD | Lifetime Intensity-weighted Days P for trend[b] (β±SE) |
|---|---|---|---|---|---|---|---|
| | Low | 17 | 1.23 ± 0.25 | | 14 | 1.17 ± 0.27 | |
| | Medium | 12 | 1.23 ± 0.54 | | 14 | 1.24 ± 0.48 | |
| | High | 13 | 1.19 ± 0.27 | 0.78 (-0.004±0.013) | 13 | 1.26 ± 0.30 | 0.75 (-0.002±0.006) |
| **Aldrin (organochlorine )** | No use | 538 | 1.19 ± 0.35 | | 538 | 1.19 ± 0.35 | |
| | Low | 106 | 1.10 ± 0.26 | | 88 | 1.07 ± 0.25 | |
| | Medium | 81 | 1.20 ± 0.31 | | 87 | 1.22 ± 0.30 | |
| | High | 75 | 1.16 ± 0.33 | 0.82 (0.001±0.006) | 87 | 1.16 ± 0.33 | 0.89 (0.0004±0.003) |
| **Carbaryl (carbamate )** | No use | 417 | 1.15 ± 0.30 | | 417 | 1.15 ± 0.30 | |
| | Low | 143 | 1.21 ± 0.34 | | 129 | 1.20 ± 0.34 | |
| | Medium | 126 | 1.20 ± 0.35 | | 130 | 1.22 ± 0.36 | |
| | High | 118 | 1.22 ± 0.32 | 0.38 (0.005±0.005) | 128 | 1.20 ± 0.32 | 0.20 (0.004±0.003) |
| **Carbofuran (carbamate)** | No use | 726 | 1.21 ± 0.38 | | 726 | 1.21 ± 0.38 | |
| | Low | 151 | 1.20 ± 0.37 | | 129 | 1.18 ± 0.39 | |
| | Medium | 111 | 1.18 ± 0.28 | | 128 | 1.18 ± 0.30 | |
| | High | 125 | 1.21 ± 0.33 | 0.99 (0.00002±0.005) | 128 | 1.22 ± 0.32 | 0.97 (0.0001±0.003) |
| **Chlordane (organochlorine)** | No use | 556 | 1.18 ± 0.33 | | 556 | 1.18 ± 0.33 | |
| | Low | 157 | 1.15 ± 0.30 | | 82 | 1.13 ± 0.29 | |
| | Medium | 28 | 1.25 ± 0.57 | | 82 | 1.16 ± 0.32 | |
| | High | 60 | 1.13 ± 0.28 | 0.06 (-0.014±0.008) | 81 | 1.18 ± 0.39 | 0.07 (-0.006±0.003) |
| **Chlorpyrifos (organophosphate)** | No use | 736 | 1.19 ± 0.35 | | 631 | 1.19 ± 0.33 | |
| | Low | 172 | 1.26 ± 0.35 | | 139 | 1.25 ± 0.35 | |
| | Medium | 158 | 1.18 ± 0.44 | | 138 | 1.16 ± 0.31 | |
| | High | 143 | 1.18 ± 0.33 | 0.66 (-0.002±0.005) | 138 | 1.22 ± 0.36 | 0.78 (0.0007±0.002) |
| **Coumaphos (organophosphate)** | No use | 974 | 1.20 ± 0.36 | | 974 | 1.20 ± 0.36 | |
| | Low | 56 | 1.26 ± 0.41 | | 38 | 1.14 ± 0.34 | |
| | Medium | 21 | 1.13 ± 0.31 | | 39 | 1.31 ± 0.43 | |
| | High | 37 | 1.17 ± 0.28 | 0.46 (-0.006±0.008) | 37 | 1.17 ± 0.28 | 0.78 (-0.001±0.004) |
| **Diazinon (organophosphate)** | No use | 608 | 1.17 ± 0.33 | | 608 | 1.17 ± 0.33 | |
| | Low | 76 | 1.20 ± 0.33 | | 65 | 1.16 ± 0.33 | |
| | Medium | 60 | 1.24 ± 0.29 | | 64 | 1.26 ± 0.34 | |

| Pesticide (class) | Pesticide Use (continuous) | Lifetime Days No. | Lifetime Days RTL Mean ± SD | Lifetime Days P for trend[a] (β± SE) | Lifetime Intensity-weighted Days No. | Lifetime Intensity-weighted Days RTL Mean ± SD | Lifetime Intensity-weighted Days P for trend[b] (β±SE) |
|---|---|---|---|---|---|---|---|
| | High | 56 | 1.18 ± 0.35 | 0.66 (0.003±0.007) | 63 | 1.19 ± 0.31 | 0.57 (0.002±0.003) |
| **Dichlorvos (DDVP, organophosphate)** | No use | 947 | 1.21 ± 0.36 | | 947 | 1.21 ± 0.36 | |
| | Low | 59 | 1.21 ± 0.38 | | 56 | 1.19 ± 0.37 | |
| | Medium | 55 | 1.17 ± 0.31 | | 55 | 1.16 ± 0.28 | |
| | High | 53 | 1.17 ± 0.28 | 0.35 (-0.005±0.005) | 55 | 1.20 ± 0.32 | 0.49 (-0.002±0.003) |
| **Dieldrin (organochlorine)** | No use | 742 | 1.18 ± 0.33 | | 742 | 1.18 ± 0.33 | |
| | Low | 41 | 1.13 ± 0.27 | | 22 | 1.22 ± 0.26 | |
| | Medium | 5 | 1.28 ± 0.39 | | 21 | 1.02 ± 0.24 | |
| | High | 17 | 1.13 ± 0.31 | 0.52 (-0.008±0.013) | 20 | 1.18 ± 0.34 | 0.78 (-0.002±0.006) |
| **Fonofos (organophosphate)** | No use | 870 | 1.21 ± 0.37 | | 870 | 1.21 ± 0.37 | |
| | Low | 95 | 1.20 ± 0.32 | | 85 | 1.16 ± 0.31 | |
| | Medium | 75 | 1.16 ± 0.31 | | 81 | 1.22 ± 0.32 | |
| | High | 79 | 1.20 ± 0.38 | 0.31 (-0.006±0.005) | 82 | 1.19 ± 0.37 | 0.41 (-0.002±0.003) |
| **Heptachlor (organochlorine)** | No use | 608 | 1.19 ± 0.35 | | 608 | 1.19 ± 0.35 | |
| | Low | 92 | 1.12 ± 0.28 | | 67 | 1.10 ± 0.30 | |
| | Medium | 62 | 1.18 ± 0.31 | | 68 | 1.14 ± 0.27 | |
| | High | 47 | 1.17 ± 0.32 | 0.76 (0.002±0.007) | 66 | 1.20 ± 0.32 | 0.96 (0.0001±0.003) |
| **Lindane (organochlorine)** | No use | 663 | 1.18 ± 0.34 | | 663 | 1.18 ± 0.34 | |
| | Low | 68 | 1.18 ± 0.40 | | 47 | 1.17 ± 0.46 | |
| | Medium | 31 | 1.15 ± 0.30 | | 46 | 1.15 ± 0.28 | |
| | High | 40 | 1.14 ± 0.29 | 0.70 (-0.003±0.007) | 46 | 1.16 ± 0.28 | 0.62 (-0.002±0.004) |
| **Malathion (organophosphate )** | No use | 265 | 1.17 ± 0.30 | | 265 | 1.17 ± 0.30 | |
| | Low | 179 | 1.17 ± 0.34 | | 181 | 1.14 ± 0.34 | |
| | Medium | 180 | 1.21 ± 0.41 | | 176 | 1.21 ± 0.41 | |
| | High | 176 | 1.17 ± 0.30 | 0.72 (-0.002±0.005) | 178 | 1.20 ± 0.29 | 0.78 (-0.001±0.002) |
| **Parathion (organophosphate )** | No use | 718 | 1.17 ± 0.32 | | 718 | 1.17 ± 0.32 | |
| | Low | 36 | 1.22 ± 0.34 | | 27 | 1.16 ± 0.29 | |
| | Medium | 17 | 1.31 ± 0.32 | | 26 | 1.30 ± 0.34 | |
| | High | 26 | 1.11 ± 0.28 | 0.72 (-0.004±0.010) | 26 | 1.15 ± 0.31 | 0.78 (-0.001±0.005) |

| Pesticide (class) | Pesticide Use (continuous) | Lifetime Days No. | RTL Mean ± SD | P for trend[a] (β± SE) | Lifetime Intensity-weighted Days No. | RTL Mean ± SD | P for trend[b] (β±SE) |
|---|---|---|---|---|---|---|---|
| **Permethrin (for crop) (pyrethroid)** | No use | 993 | 1.20 ± 0.37 | | 993 | 1.20 ± 0.37 | |
| | Low | 62 | 1.23 ± 0.33 | | 37 | 1.24 ± 0.34 | |
| | Medium | 19 | 1.23 ± 0.39 | | 36 | 1.15 ± 0.34 | |
| | High | 28 | 1.21 ± 0.33 | 0.69 (0.004±0.010) | 36 | 1.28 ± 0.32 | 0.50 (0.003±0.004) |
| **Phorate (organophosphate )** | No use | 537 | 1.19 ± 0.34 | | 537 | 1.19 ± 0.34 | |
| | Low | 113 | 1.15 ± 0.31 | | 91 | 1.15 ± 0.33 | |
| | Medium | 75 | 1.13 ± 0.29 | | 92 | 1.15 ± 0.30 | |
| | High | 85 | 1.16 ± 0.30 | 0.46 (-0.005±0.006) | 90 | 1.16 ± 0.27 | 0.58 (-0.002±0.003) |
| **Terbufos (organophosphate)** | No use | 695 | 1.20 ± 0.39 | | 695 | 1.20 ± 0.39 | |
| | Low | 195 | 1.20 ± 0.32 | | 141 | 1.19 ± 0.33 | |
| | Medium | 99 | 1.19 ± 0.32 | | 140 | 1.21 ± 0.32 | |
| | High | 129 | 1.21 ± 0.32 | 0.76 (0.001±0.005) | 140 | 1.21 ± 0.30 | 0.59 (0.001±0.002) |
| **Fumigants** | | | | | | | |
| **Aluminum phosphide (inorganic fumigant)** | No use | 780 | 1.17 ± 0.33 | | 780 | 1.17 ± 0.33 | |
| | Low | 16 | 1.16 ± 0.17 | | 11 | 1.23 ± 0.33 | |
| | Medium | 13 | 1.16 ± 0.17 | | 11 | 1.08 ± 0.13 | |
| | High | 3 | 1.21 ± 0.35 | 0.82 (0.005±0.025) | 10 | 1.19 ± 0.24 | 0.75 (0.003±0.010) |
| **Methyl bromide (bromomethane, Brom_O_Gas)** | No use | 1034 | 1.20 ± 0.33 | | 1034 | 1.20 ± 0.33 | |
| | Low | 66 | 1.17 ± 0.31 | | 62 | 1.17 ± 0.33 | |
| | Medium | 70 | 1.21 ± 0.37 | | 61 | 1.25 ± 0.61 | |
| | High | 49 | 1.33 ± 0.71 | 0.96 (-0.0004±0.007) | 61 | 1.26 ± 0.44 | 0.65 (-0.002±0.004) |
| **Ethylene dibromide (brominated )** | No use | 767 | 1.17 ± 0,31 | | 767 | 1.17 ± 0.31 | |
| | Low | 20 | 1.17 ± 0.59 | | 14 | 1.21 ± 0.66 | |
| | Medium | 7 | 1.19 ± 0.37 | | 14 | 1.22 ± 0.37 | |
| | High | 13 | 1.51 ± 0.67 | 0.55 (0.009±0.016) | 12 | 1.45 ± 0.73 | 0.79 (0.002±0.007) |
| **Carbon tetrachloride/ Carbon disulfide–80/20 mix** | No use | 734 | 1.18 ± 0.33 | | 734 | 1.18 ± 0.33 | |
| | Low | 33 | 1.24 ± 0.45 | | 24 | 1.28 ± 0.50 | |
| | Medium | 19 | 1.07 ± 0.20 | | 25 | 1.11 ± 0.23 | |
| | High | 21 | 1.07 ± 0.38 | 0.06 (-007±0.006) | 23 | 1.05 ± 0.36 | 0.10 (-0.003±0.003) |
| **Fungicides** | | | | | | | |
| **Benomyl (benzimidazole)** | No use | 743 | 1.17 ± 0.33 | | 743 | 1.17 ± 0.33 | |

| Pesticide (class) | Pesticide Use (continuous) | Lifetime Days No. | Lifetime Days RTL Mean ± SD | Lifetime Days P for trend[a] (β± SE) | Lifetime Intensity-weighted Days No. | Lifetime Intensity-weighted Days RTL Mean ± SD | Lifetime Intensity-weighted Days P for trend[b] (β±SE) |
|---|---|---|---|---|---|---|---|
| | Low | 21 | 1.24 ± 0.37 | | 21 | 1.22 ± 0.37 | |
| | Medium | 21 | 1.17 ± 0.32 | | 19 | 1.24 ± 0.34 | |
| | High | 17 | 1.32 ± 0.28 | 0.68 (0.005±0.012) | 19 | 1.25 ± 0.28 | 0.50 (0.004±0.006) |
| **Captan (phthalimide)** | No use | 968 | 1.21 ± 0.36 | | 968 | 1.21 ± 0.36 | |
| | Low | 58 | 1.15 ± 0.33 | | 39 | 1.15 ± 0.33 | |
| | Medium | 20 | 1.14 ± 0.26 | | 38 | 1.12 ± 0.31 | |
| | High | 38 | 1.18 ± 0.33 | 0.69 (-0.003±0.009) | 38 | 1.20 ± 0.33 | 0.24 (-0.006±0.005) |
| **Chlorothalonil (halogenated benzonitrile)** | No use | 1132 | 1.20 ± 0.35 | | 1132 | 1.20 ± 0.35 | |
| | Low | 34 | 1.19 ± 0.41 | | 27 | 1.22 ± 0.42 | |
| | Medium | 27 | 1.19 ± 0.30 | | 28 | 1.14 ± 0.32 | |
| | High | 20 | 1.27 ± 0.41 | 0.27 (0.010±0.009) | 26 | 1.28 ± 0.37 | 0.39 (0.004±0.005) |
| **Maneb/Mancozeb (ethylenebisdithiocarbamate)** | No use | 737 | 1.17 ± 0.32 | | 737 | 1.17 ± 0.32 | |
| | Low | 28 | 1.32 ± 0.57 | | 21 | 1.17 ± 0.36 | |
| | Medium | 19 | 1.19 ± 0.31 | | 20 | 1.42 ± 0.62 | |
| | High | 14 | 1.22 ± 0.42 | 0.45 (0.008±0.010) | 20 | 1.19 ± 0.35 | 0.72 (0.002±0.005) |
| **Metalaxyl (acylalanine)** | No use | 675 | 1.17 ± 0.31 | | 675 | 1.17 ± 0.31 | |
| | Low | 41 | 1.14 ± 0.29 | | 40 | 1.11 ± 0.29 | |
| | Medium | 49 | 1.26 ± 0.35 | | 40 | 1.23 ± 0.30 | |
| | High | 30 | 1.27 ± 0.61 | 0.61 (0.005±0.010) | 39 | 1.32 ± 0.57 | 0.76 (0.001±0.005) |

[a]P-value of linear regression coefficient for the natural logarithm of RTL (continuous) regressed on natural logarithm of lifetime-days of pesticide use (continuous), adjusted for age at buccal collection (continuous), state (IA vs. NC), license types (private vs. commercial), use of chewing tobacco regularly for six months or longer (yes vs. no), total pesticide-exposure days (continuous). Regression coefficient and standard error of the regression coefficient provide under the p-value. RTL mean by tertile of lifetime-days of pesticide exposure is the RTL arithmetic mean of each tertile along with the standard deviation (SD) of each tertile.

[b]P-value of linear regression coefficient for the natural logarithm of RTL (continuous) regressed on natural logarithm of lifetime-intensity-weighted days of pesticide use (continuous), adjusted for age at buccal collection (continuous), state (IA vs. NC), license types (private vs. commercial), use of chewing tobacco regularly for six months or longer (yes vs. no)), total pesticide-exposure days (continuous). Regression coefficient and standard error of the regression coefficient provide under the p-value. RTL mean by tertile of lifetime-intensity-weighted days of pesticide exposure is the RTL arithmetic mean of each tertile along with the standard deviation (SD) of each tertile.

**Figure S1. Standard curve in every T and S PCR run**. Two representative plots of standard curves for the Tel and Hgb reactions are shown. We note that the reactions were standardized on a ABI 7900HT system using a high precision Hamilton Starlet 8-channel liquid handler to limit sample to sample and plate to plate variability. As shown in the plots, this approach allowed to obtain optimal calibration of the reactions through the standard curves.

*(A) T reaction*

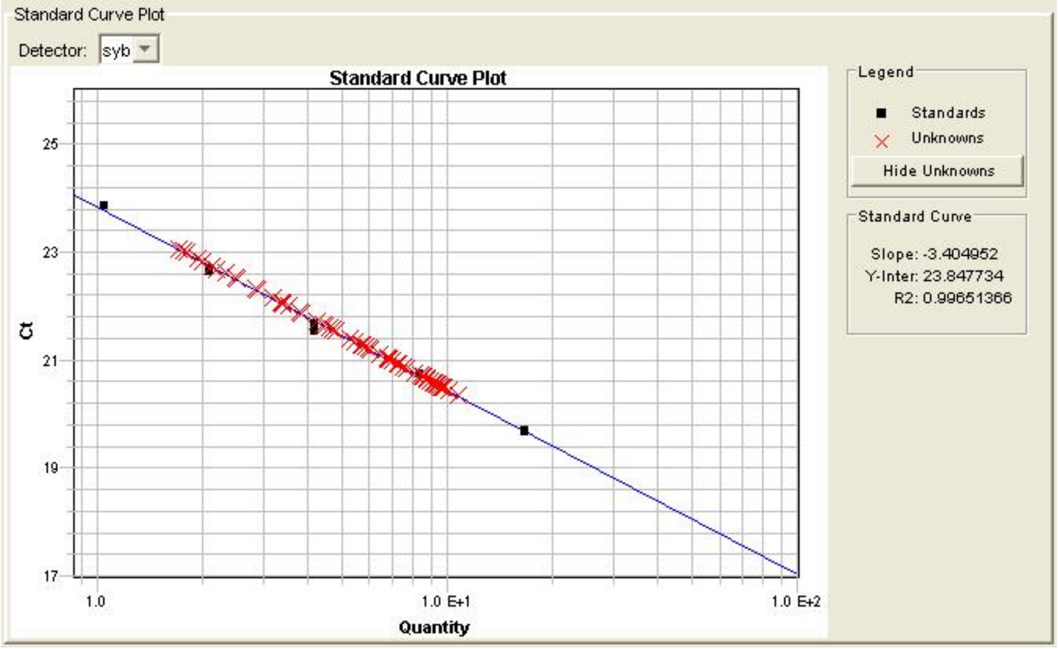


*(B) Hgb reaction*

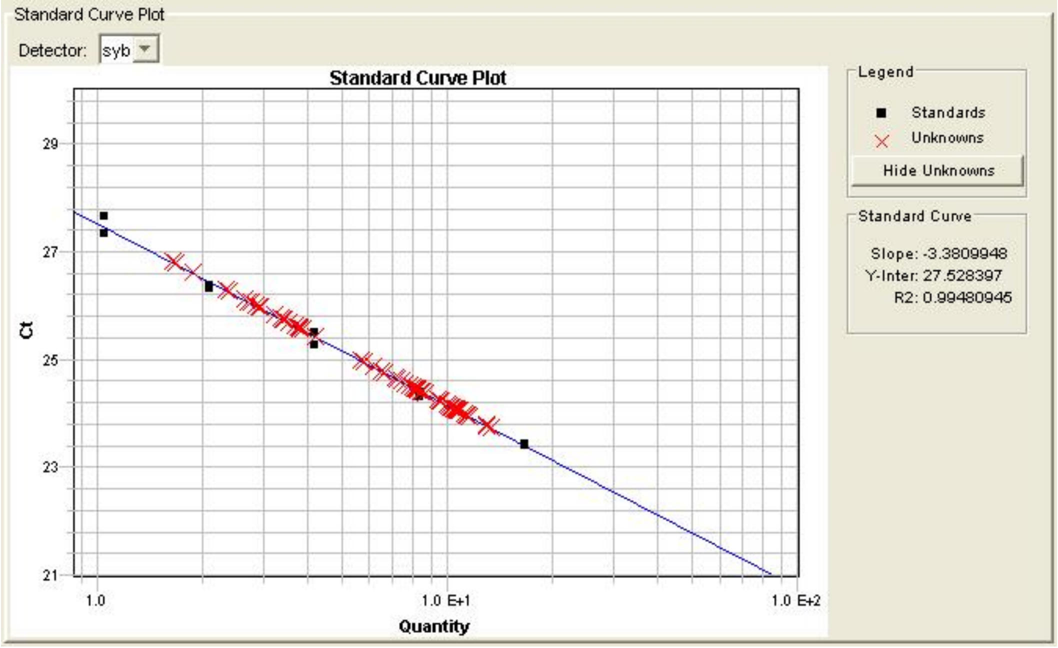

Supplement: (565 KB) PDF [file ehp.1206432.s001.pdf]
